# Negative Emotion Weakens the Degree of Self-Reference Effect: Evidence from ERPs

**DOI:** 10.3389/fpsyg.2016.01408

**Published:** 2016-09-28

**Authors:** Wei Fan, Yiping Zhong, Jin Li, Zilu Yang, Youlong Zhan, Ronghua Cai, Xiaolan Fu

**Affiliations:** ^1^Cognition and Human Behavior Key Laboratory of Hunan Province, Hunan Normal UniversityChangsha, China; ^2^State Key Laboratory of Brain and Cognitive Science, Institute of Psychology (CAS)Beijing, China

**Keywords:** self-reference processing, negative emotion, degree of self-reference effect, N2, P3

## Abstract

We investigated the influence of negative emotion on the degree of self-reference effect using event-related potentials (ERPs). We presented emotional pictures and self-referential stimuli (stimuli that accelerate and improve processing and improve memory of information related to an individual’s self-concept) in sequence. Participants judged the color of the target stimulus (self-referential stimuli). ERP results showed that the target stimuli elicited larger P2 amplitudes under neutral conditions than under negative emotional conditions. Under neutral conditions, N2 amplitudes for highly self-relevant names (target stimulus) were smaller than those for any other names. Under negative emotional conditions, highly and moderately self-referential stimuli activated smaller N2 amplitudes. P3 amplitudes activated by self-referential processing under negative emotional conditions were smaller than neutral conditions. In the left and central sites, highly self-relevant names activated larger P3 amplitudes than any other names. But in the central sites, moderately self-relevant names activated larger P3 amplitudes than non-self-relevant names. The findings indicate that negative emotional processing could weaken the degree of self-reference effect.

## Introduction

Self-reference can increase the speed and quality of processing and memorization of information related to the individual’s self-concept (Rogers et al., 1977; Kim, 2012). Rogers et al. (1977) confirmed that the self-relevant stimulus information is associated with memory, observing that subjects performed better on memory tasks concerning self-relevant stimuli information. Hence, Rogers et al. (1977) put forward the concept of the memory advantage aspect of the self-reference effect.

Many studies have focused on the types of the self. The same type of self-referential processing should involve the same neural mechanisms (Berlad and Pratt, 1995; Symons and Johnson, 1997; Ninomiya et al., 1998; Miyakoshi et al., 2007; Zhao et al., 2011; Schneider et al., 2012; Winter et al., 2015). For example, some researchers have used self-limb processing (Feinberg, 1997; Meador et al., 2000; Ehrsson et al., 2004), face recognition (Turk et al., 2002; Platek et al., 2006), and hand recognition (Su et al., 2010; Ferri et al., 2012) to explore the physical self. Others have used individuals’ own voices (Kaplan et al., 2008), handwriting (Chen A. et al., 2008), personal characteristics (Craik et al., 1999; Kelley et al., 2002; Serino et al., 2008; Schneider et al., 2012), and autobiographical memory (Gray et al., 2004; Summerfield et al., 2009; Kim, 2012) to explore the psychological self. Research on self-referential processing can vary considerably in stimulus materials and methods. Different stimulus materials can elicit different levels of self-referential processing and may activate different neural mechanisms (Gillihan and Farah, 2005). Although self-concept is represented at multiple levels, the psychological self is considered the core aspect of self (Northoff et al., 2006).

Studies showed that self-categories should include the individual self and the collective self. Individual self refers to one’s own status, characteristics, behavior, and other personal characteristics (such as being optimistic, smart, and diligent), while collective self refers to the membership in specific groups, social contacts, and social status (Northoff et al., 2006; Gaertner et al., 2012). A series of studies have shown the existence of the collective self-reference effect. For example, in a previous study (Johnson et al., 2002), participants associated with the same university or in the same family were found to experience collective self-relevant stimuli. Collective self-related information showed a stronger association with memory than non-collective self-related information (Wagar and Cohen, 2003). Zhang et al. (2006) completed an fMRI study with an auto-correlation pattern. Their results showed that Chinese mother-references elicited a response in the medial prefrontal cortex (mPFC), which is consistent with the results of self-reference experiments. Zhang et al. (2006) deduced that the mother is a part of the Chinese collective self in Chinese culture. Zhao et al. (2009) studied the individual self-reference effect and collective self-reference effect and found that P300 amplitude elicited by the collective self-relevant stimulus was larger than those elicited by familiar and unfamiliar stimuli.

However, the studies reviewed above focused on categorical differences, considering self-relevant effects as behavioral or neural activation differences between self-relevant and non-self-relevant stimuli. The previous studies may have failed to take into account the degree of self-relevance. In real-life situations, stimuli with different levels of relevance to self are often different in adaptive significance, with highly self-relevant stimuli possessing greater biological and social significance than minimally self-relevant stimuli. That is why, for example, hearing one’s own name may result in more attention than hearing a friend’s name, although both names may elicit more attention than non- self-relevant names. These different levels of responsiveness in turn may be reflected in measurable differences in brain activity. Fan et al. (2013) put forward the concept of the degree of self-reference effect on the basis of self-reference effect, which refers to the degree to which stimuli that are more self-relevant are processed faster and more precisely than those that are less self-relevant. There are some studies that have either studied or relied upon this concept – the degree of self-reference effect. Keyes et al. (2010) explored the mechanism by which participants processed images of their own faces, their friends’ faces, and strangers’ faces. Tacikowski et al. (2011) investigated the effect of repetition on the processing of names and faces. They also investigated the pattern of brain activation during the recognition of full names of the target stimulus persons. Results showed that participants learned faces more readily than names, possibly because faces carry more semantic information. This pattern of results supported the existence of a role for mPFC in the processing of personally relevant information, irrespective of information modality.

Chen et al. (2011) directly investigated the self-relevant degree effect, and found that higher self-relevant stimuli elicited enhanced P3 amplitudes. Chen et al. (2011) observed that one’s own name is the most specific descriptor of the self. One’s province is more general but still more specific than country (e.g., China or America, which are equal in generality). Chen et al. (2011) found that larger P3 amplitudes were elicited by the participants’ names followed by the names of their home provinces, but they found no differences in P3 amplitudes between the names “China” and “America.” For this reason, it was here that it is possible that P3 was modulated by the degree of specificity rather than the degree of self-relevance. A similar effect was also observed in a previous study (Fan et al., 2013). Highly self-relevant names elicited larger P3 mean amplitudes than the moderately self-relevant names, which, in turn, yielded larger P3 values than the minimally self-relevant names. Minimally self-relevant stimuli elicited larger P3 mean amplitudes than non-self-relevant stimuli. In summary, studies that used stimuli with different degrees of self-relevance to investigate the degree of self- reference effect, either directly or indirectly, produced results that were more precise and reliable than those of studies that compared self-relevant stimuli to non-self-relevant stimuli.

Other researchers have obtained similar findings for valence strength in the study of emotion. Although emotional stimuli can attract more attention than neutral stimuli, extreme emotional stimuli always induce more brain attention bias than medium-level emotional stimulation (Yuan et al., 2007, 2008b). Additional research has shown that, unlike slightly negative emotions, highly negative emotions affect the lives of individuals to a great degree. Highly negative emotions affect individuals’ memory profoundly, impede creativity, and predispose the individual to unwise decisions (Watkins et al., 1996; Coon and Mitterer, 2010). There are similarities between self-relevant stimuli and emotional stimuli, especially in physiological fields. Other investigations indicate that the processing of self-relevant stimuli and emotional stimuli can activate similar neural machinery, such as the nucleus accumbens, insula, or ventral mPFC (Phan et al., 2004; Taylor and Fragopanagos, 2005). Attribution research demonstrates that the processing of self-referential stimuli and that of emotional stimuli have reciprocal effects on each other. Watson et al. (2007), for instance, used an autocorrelation paradigm to record the EEG of subjects when they judged the emotional words, and found self-referential information processing to be highly correlated with emotional valence on the N400, which demonstrated that they are not independent.

Watson et al. (2008) compared the judgment result of self-reference about emotional words between depressed subjects and normal subjects, and the results support the existence of the self-positivity bias in non-depressed individuals. Depressed individuals were able to accurately identify the emotional valence of the word stimuli, but failed to associate this emotional valence with self-reference. Herbert et al. (2011a,b) determined at which temporal processing stages self-other discrimination in emotion processing occurs, and the results support the conclusion that, for verbal emotional stimuli, self-other discrimination first occurs at higher-order, cortical processing stages.

In addition, the processing of self-referential stimuli and of emotional stimuli has been shown to have several similarities. Such stimuli activate similar neural mechanisms and can elicit the same composition of event-related potentials (ERPs). A large number of studies have found that emotional stimuli are associated with variations in P3 (e.g., Ito et al., 1998; Ito and Cacioppo, 2000; Huang and Luo, 2006). Emotional stimuli frequently elicit larger P3 amplitudes than neutral stimuli. Previous studies have shown that self-referential stimuli frequently elicited larger P3 amplitudes than the control stimuli (Ninomiya et al., 1998; Miyakoshi et al., 2007; Knyazev, 2013, 2014). In previous works, results showed similar effects. In one study (Zhao et al., 2009), results showed that the participant’s own name and the name of his or her alma mater elicited larger P3 amplitude than the names of other schools. Another study (Fan et al., 2011) used the subject’s own national flag as self-referential stimulus, which elicited larger P3 amplitudes than flags of other nations. Because emotions elicit similar ERPs components and have overlapping neural mechanisms with self-referential processing, we hypothesized that emotions have an important effect on the degree of self-reference effect.

Previous studies demonstrated that the P3 component might also be linked to the degree of self- reference effect. Results showed that more highly self-relevant stimuli elicited larger P3 amplitudes. Self-referential stimuli underwent more sophisticated processing in P3 stage (Chen et al., 2011; Fan et al., 2013). If emotional stimuli have a significant impact on the degree of self-reference effect, P3 amplitudes undergo significant changes. In a recent study, Zhong et al. (2014) examined the effects of positive emotion on the degree of self-reference effect and found no significant impact. The processing of positive emotion and self-reference may be independent of each other. The processing of individual positive emotion has been shown to be different from that of negative emotion. For instance, Pourtois et al. (2004) found that after fearful facial expressions became visible, the presence of a stimulus in the same position elicited larger P1 amplitude, but happy expressions did not. Pourtois et al. (2006) used a similar paradigm to conduct an fMRI study. That study showed that fearful facial expressions elicited larger activation than happy expressions in the bilateral temporal parietal and right occipital parietal areas. The evidence reported by Pourtois et al. (2006) suggests that the influence of negative emotion is different from that of positive emotion on the degree of self-reference effect. Because negative emotion can attract more attention, negative emotional processing can weaken the degree of self-reference effect.

## Materials and Methods

### Participants

Eighteen paid volunteers, which all undergraduate or postgraduate students, (nine women, nine men) aged 19–24 years (mean age, 22) participated in the experiment. All subjects were healthy, right-handed, had normal or corrected vision, and reported no history of cerebral injury.

### Apparatus and Stimuli

The stimuli were displayed on 17-inch cathode-ray tube (CRT) monitors with 75 Hz refresh rate and a screen resolution of 1024 pixels × 768 pixels. The software package E-prime 2.0 was used for stimuli presentation and data collection.

Negative and neutral emotional pictures served as stimuli; self-relevant names with different levels served as target stimuli. Because a cultural bias for the International Affective Picture System (IAPS) has been reported in Chinese individuals (Huang and Luo, 2004), we selected pictures from the native Chinese Affective Picture System (CAPS; Bai et al., 2005) as the priming stimuli. Here, we selected a total of 120 emotional pictures (60 negative pictures and 60 neutral pictures) from the CAPS, based on the valence, arousal, and familiarity scores provided in the database.

To verify that the published values for the pictures indeed differed as we intended, we conducted an analyses of variance (ANOVA) on valence, arousal, and familiarity. Negative pictures have a significant lower mean valences (*M* = 2.46, *SD* = 0.27) than neutral pictures (*M* = 5.14, *SD* = 0.11) [*F*(1,59) = 3.38, *p* < 0.05]. Mean arousal also differed significantly [*F*(1,59) = 2.96, *p* < 0.05] between negative pictures (*M* = 5.96, *SD* = 0.76) and neutral pictures (*M* = 4.01, *SD* = 0.97).

However, familiarity of neutral and negative pictures was statistically equivalent [*F*(1,59) = 0.41, *p* > 0.05]. These results indicated that in negative pictures, there was no difference in valence [*F*(1,59) = 0.33, *p* > 0.05], familiarity [*F*(1,59) = 0.47, *p* > 0.05], or arousal level [*F*(1,59) = 0.65, *p* > 0.05]; likewise no differences were observed for the neutral pictures in valence [*F*(1,59) = 0.27, *p* > 0.05], familiarity [*F*(1,59) = 0.19, *p* > 0.05] and arousal level [*F*(1,59) = 0.11, *p* > 0.05].

According to recent studies (Chen et al., 2011; Fan et al., 2013), amplitudes elicited by minimally self-referential stimuli and familiar stimuli showed no significant differences. We presented self-referential stimuli divided by relevance into three categories: (a) those with high correlations to self-material, which are participants’ names; (b) those moderately correlated with self-material, which are names of each participants’ father; and (c) familiar stimuli that do not have a sense of connection to the participant, which is the name of a foreign head of state. We present stimuli from each class of self-referential stimulus 120 times (60 times in blue font and 60 times in green font). In order to conceal the true purpose of the experiment, subjects were asked to perform an irrelevant color discrimination task.

### Procedure

#### Behavioral Task

**FIGURE 1:**
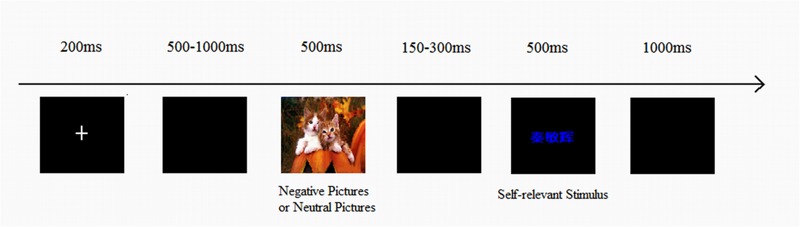
**The sequence of events in an experimental trial**.

In each trial, a fixation cross was presented for 200 ms, followed by a black screen presented for a random interval between 500 and1000 ms. A priming stimulus then appeared for 500 ms, followed by a black screen presented for a random interval between 150 and 300 ms. A target stimulus then appeared for 500 ms, followed by a black screen presented for 1000 ms. (see **Figure 1**) schematically depicts the sequence of events in a typical trial).

The subjects’ task was to judge the color of the target stimulus. If the target stimulus was presented in blue, participants were to press the “1” key; if the target stimulus was presented in green, they were press the “2” key. The experiment consisted of 10 practice trials. The formal experiment consists of 360 trials, divided into three blocks of 120 trials each. The 120 emotionally significant pictures selected from the CAPS served as priming stimuli. Each was presented once to participants within a single block; each block contained 60 neutral pictures and 60 negative pictures. Priming stimulus and target stimulus were presented in sequence. Then the next trial began.

Following the color discrimination task, in order to assess perceived stimulus familiarity and self-relevance of each stimulus, participants rated both variables, using a 9-point self-report (self-relevance: 1 = “not self-related at all” to 9 = “extremely self-related”; familiarity: 1 = “not familiar at all” to 9 = “extremely familiar”), with the order of the two rating tasks counterbalanced across participants. The purpose of the ratings was to confirm that the self-relevance of the stimulus materials used differed ordinally and monotonically (e.g., high self-relevance > moderate self-relevance > not self-relevant) and that familiarity was statistically equivalent across stimulus classes.

#### EEG Recording

We recorded continuous electroencephalograms (EEGs) using 64 scalp silver/silver-chloride electrodes placed using the international 10–20 system. All electrodes were referenced to an electrode at the left mastoid and re-referenced off-line to the bilateral mastoid (Petten et al., 2005). We recorded horizontal electro oculograms (EOG) in a bipolar manner from two electrodes placed 1.5 cm lateral to the left and right outer canthi, and vertical EOGs (VEOG) from electrodes below and above the left eye. The impedance of each electrode was kept below 5 kΩ. EEG signals were amplified (half-amplitude band pass 0.05–40 Hz) and digitized at a sampling rate of 500 Hz.

#### ERP Data Processing and Statistics

Event-related potentials recorded under each stimulus condition were averaged separately off-line with epochs beginning an average of 100 ms prior to and ending 500 ms after the onset of the stimulus. We excluded any trials affected by eye blinks (VEOG exceeding ± 50 μV relative to baseline) or other artifacts (a voltage exceeding ± 50 μV at any electrode location relative to baseline), because we considered such trials as contaminated Previous studies have suggested a lateralization of visual self-recognition (Turk et al., 2002; Uddin et al., 2005; Ma and Han, 2010), we examined the caudality and laterality effects, following 15 electrode sites for statistic analysis according to the previous study(Chen et al., 2015),F3,FC3,C3,CP3,P3(five left sites); Fz, FCz, Cz, CPz, Pz (five midline sites); and F4,FC4,C4,CP4,P4 (five right sites) are selected. Prominent P1 (150–250 ms), N2 (250–350 ms), the mean amplitude of P3 (350–500 ms) components were elicited during all two conditions.

### Behavioral Results

The ratings after the experiment showed a significant main effect of self-related stimuli, *F*(2,38) = 26.12, *p* < 0.001. *Post hoc* comparisons with Bonferroni correction showed that highly self-related names had greater rating scores than moderately self-related names and non-self names, all *t*(19) > 4.28, all *p* < 0.05. Moderately self-related names had higher rating scores than non-self names, *t*(19) = 7.91, *p* < 0.01. However, the three types of self-related stimuli showed no significant difference in familiarity, *F*(2,38) = 1.15, *p* > 0.05. We concluded, therefore, that the stimuli we chose as high, medium, and low self-related and as familiar, based on published values, were in fact effective in the empirical results for the present study.

The participants performed the irrelevant color discrimination task, which was unrelated to the experimental goal. Consequently, we did not analyze the accuracy rates and response times.

### Priming Stimuli ERPs Results

Separate four-way repeated measures analyses of variance (ANOVAs) were conducted for the mean amplitude of each component. ANOVA factors were stimulus type (three levels: highly self-relevant, moderately self-relevant, and non-self-relevant), valence (two levels: negative, neutral), laterality (three levels: left, midline, and right), and caudality (five levels: front, front-central, central, central-parietal, and parietal sites). The degrees of freedom of the *F*-ratios were corrected with the help of the Greenhouse-Geisser method.

#### Mean Amplitude for N1 (50–150 ms)

ANOVAs on N1 (50–150 ms) mean amplitude [*F*(1,17) = 1.02, *p* > 0.05] demonstrated no significant effect.

#### Mean Amplitude for P2 (150–250 ms)

For the P2 mean amplitude, a multiple ANOVA showed a significant interaction between emotional type and caudality [*F*(4,68) = 10.82, *p* < 0.001]. The simple effects analysis showed negative emotional stimuli elicited larger P2 amplitudes than neutral condition in front-central sites [*F*(1,17) 285 = 3.72, *p* < 0.05], central sites [*F*(1,17) = 8.15, *p* < 0.01], central-parietal sites [*F*(1,17) = 10.43, *p* < 0.01] and parietal sites [*F*(1,17) = 19.12, *p* < 0.001].

### Target Stimulus ERPs Results

**FIGURE 2:**
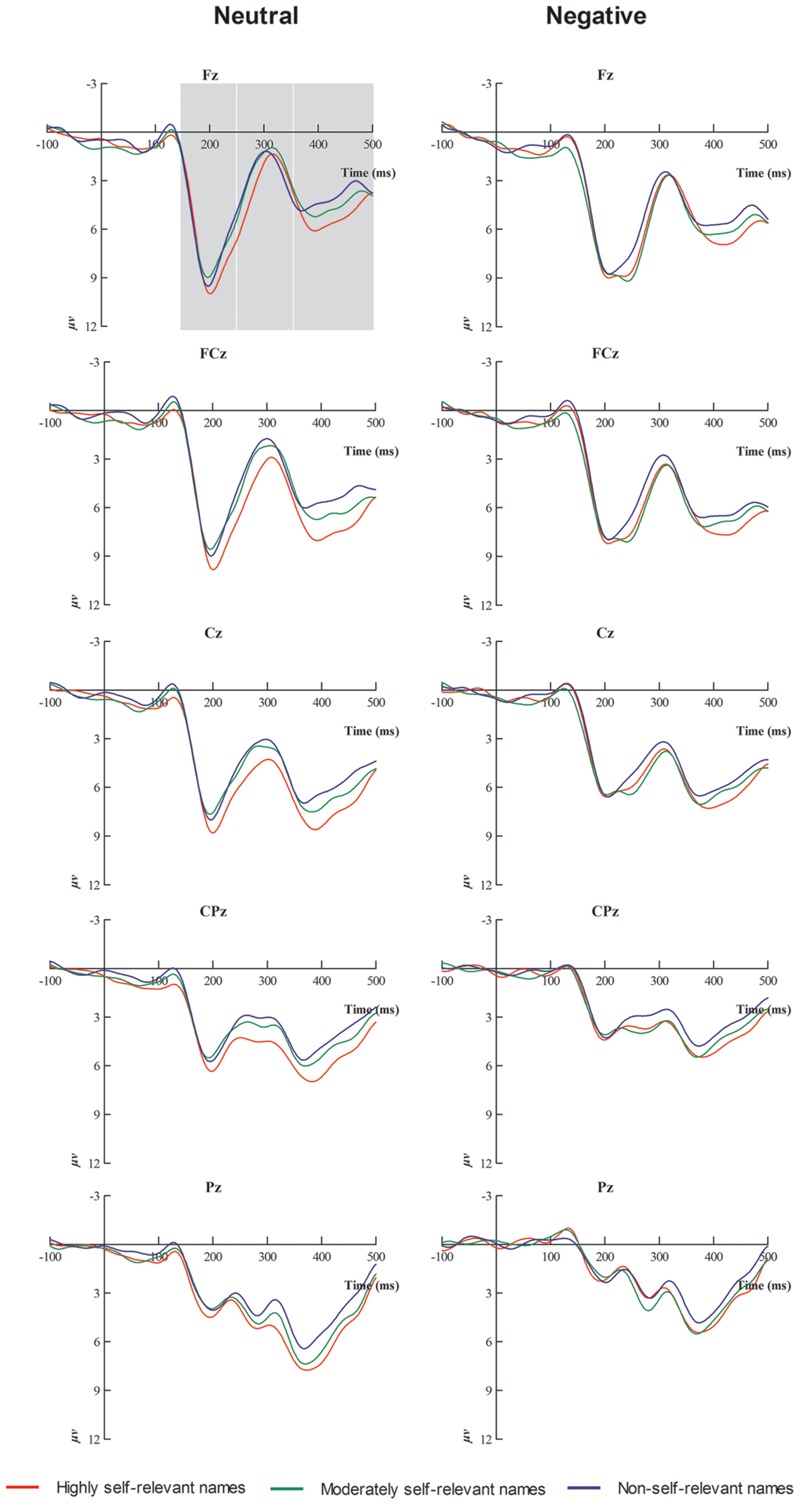
**Averaged event-related potentials (ERPs) at Fz, Fcz, Cz, Cpz, and Pz for highly self-relevant, moderately self-relevant, and non-self-relevant stimulus under neutral and negative emotional conditions**.

**FIGURE 3:**
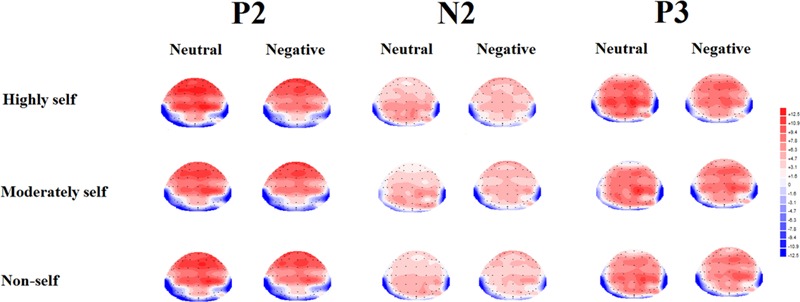
**Topographical maps of the voltage amplitudes (highly self-relevant, moderately self-relevant and non-self-relevant) in P2 (150–250 ms), N2 (250–350 ms), and P3 (350–500 ms) under neutral and negative emotional condition**.

As shown in **Figures 2** and **3**, N1, P2, N2, and P3 components were elicited under each of the three stimulus conditions. ANOVAs on N1 (50–150 ms) mean amplitude [*F*(2,34) = 0.39, *p* > 0.05] demonstrated no significant effects.

#### Mean Amplitude for P2 (150–250 ms)

For the P2 mean amplitude, results showed that there was a significant effect of emotion type, and neutral emotional conditions elicited larger P2 mean amplitude than negative emotional conditions [*F*(1,17) = 11.5, *p* < 0.01]. There was a significant interaction between emotional type and caudality [*F*(4,68) = 17.23, *p* < 0.001]. Simple effects analysis showed that, under negative emotional priming conditions, the target stimulus elicited smaller P2 mean amplitudes than the neutral condition in central sites [*F*(1,17) = 10.5, *p* < 0.01], central-parietal sites [*F*(1,17) = 21.18, *p* < 0.001] and parietal sites [*F*(1,17) = 32.1, *p* < 0.001].

#### Mean Amplitude for N2 (250–350 ms)

For the N2 mean amplitude, there was a significant interaction between emotional type and self-referential type [*F*(2,34) = 5.58, *p* < 0.05]. The simple effects analysis showed that, under neutral conditions, highly self-relevant names elicited smaller N2 mean amplitudes than other names [all *Fs*(1,17) > 5.58, all *p*s < 0.05]. Highly and moderately self-referential names elicited smaller N2 mean amplitudes under negative emotional priming conditions than under neutral conditions [all *F*s(1,17) > 4.63, all *p*s < 0.05]. ANOVA revealed a significant interaction between emotional type and caudality [*F*(4,68) = 54.15, *p* < 0.001]. Simple effects analysis showed that, under negative emotional priming conditions, the target stimulus elicited smaller N2 mean amplitudes at frontal sites than under neutral conditions [*F*(1,17) = 37.25, *p* < 0.001], front-central sites [*F*(1,17) = 21.05, *p* < 0.001], central-parietal sites [*F*(1,17) = 14.76, *p* < 0.01], and parietal sites [*F*(1,17) = 40.09, *p* < 0.001]. ANOVA also revealed marginally significant interaction between self-referential type and caudality [*F*(8,136) = 2.78, *p* = 0.06]. The simple effect analysis showed that highly self-relevant names elicited smaller N2 mean amplitude than other names in central sites [all *F*s(1,17) > 3.42, all *p*s < 0.05] and central-parietal sites [all *F*s(1,17) > 5.36, all *p*s < 0.01]. ANOVA also revealed a significant interaction between emotional type and laterality [*F*(2,34) = 3.66, *p* < 0.05]. The simple effect analysis showed that target stimulus under negative emotional conditions elicited smaller N2 mean amplitude than under neutral condition in midline sites [*F*(1,17) = 11.51, *p* < 0.01]. In addition, a multiple ANOVA also revealed a significant interaction between self-referential type and laterality [*F*(4,68) = 3.96, *p* < 0.05]. The simple effect analysis showed that highly self-relevant name elicited smaller N2 mean amplitude than other names in midline sites [all *F*s(1,17) > 5.2, all *p*s < 0.05].

#### Mean Amplitude of P3 (350–500 ms)

For the P3 mean amplitude, ANOVA revealed a significant main effect of emotional type. The target stimulus elicited larger P3 mean amplitudes under neutral condition than under negative emotional conditions [*F*(1,17) = 3.53, *p* < 0.05]. Furthermore, there was a significant main effect of self- referential type [*F*(2,34) = 3.73, *p* < 0.05]. *Post hoc* multiple comparisons revealed that highly self- relevant names elicited larger P3 mean amplitude than other names, while moderately self-relevan names elicited larger P3 mean amplitudes than non-self-relevant names [all *F*s(1,17) > 3.94, all *p*s < 0.05]. ANOVA also indicated a significant interaction between emotional type and caudality [*F*(4,68) = 30.15, *p* < 0.001]. In the simple effect analysis under negative emotional priming conditions, the target stimulus elicited smaller P3 mean amplitudes than under neutral condition in frontal sites [*F*(1,17) = 10.85, *p* < 0.01], central-parietal sites [*F*(1,17) = 18.23, *p* < 0.01], and parietal sites [*F*(1,17) = 25.54, *p* < 0.001]. In addition, ANOVA revealed a significant interaction between self- referential type and laterality [*F*(4,68) = 5.63, *p* < 0.01]. The simple effect analysis indicated that highly self-relevant names elicited larger P3 mean amplitudes than other names in left and midline sites [all *F*s(1,17) > 3.68, all *p*s < 0.05], while moderately self-relevant names elicited larger P3 mean amplitudes than non-self-relevant names in midline sites [all *F*s(1,17) > 5.31, all *p*s < 0.05].

## Discussion

The present study identified the degree of self-reference effect at the implicit level, and results showed that different emotions affect the degree of self-reference effect. The interaction between emotion and caudality in P2 is consistent with the previous studies about emotion (Chen J. et al., 2008; Yuan et al., 2008a). The results of the present study showed no significant difference among the three kinds of self- referential stimuli in terms of N1 mean amplitudes under negative and neutral emotional conditions. That outcome may be due to equilibrium of physical properties such as size, length, and complexity of the stimulus. In the early phase (50–150 ms), the presentation of negative and neutral emotional pictures did not affect the subjects’ self-referential stimuli processing, and it is estimated that this effect may occur during later stages. Self-referential processing elicited larger P2 amplitudes under neutral conditions than under negative emotional conditions. Evidence indicated that frontal P2 activity is indicative of rapid detection of typical stimulus features (Karayanidis and Michie, 1996; Thorpe et al., 1996). In addition, a study indicated that P2, an attention- related component, had larger amplitudes and shorter latencies in response to emotional stimuli than in response to neutral stimulus, and stimuli of greater biological importance drew attention more easily (Carretié et al., 2001). However, this attention lacked advanced cognitive processing and allocation of control resources (Del Cul et al., 2007; Hu et al., 2011). As the negative pictures occupy more attentional resources (Chen J. et al., 2008; Yuan et al., 2008a) and leave limited resources to process later self-referential stimulus, self-referential processing by participants subjected to negative priming conditions showed smaller P2 amplitudes than under neutral conditions.

Under neutral conditions, highly self-relevant names elicited smaller N2 amplitudes than other names. Existing research has suggested that self-referential stimulus elicited smaller N2 amplitudes than other stimuli (Folstein and Van Petten, 2008). This previous study showed that N2 can usually be described as a non-specific component that corresponds to an attention-switching mechanism and that it is followed by a positive P300 wave (Kiehl et al., 2001). Näätänen et al. (1982) observed, that under passive conditions, an N2 component occurred when the stimulus was salient enough to trigger a switch of attention.

The results suggested that highly self-relevant names might automatically capture attention, even if they were not targets. And results also showed that highly self-relevant names evoked shorter amplitudes N2 than other names, which may be associated with the better psychological saliency and biological importance of highly self-relevant names, leading to easier recognition and requiring fewer cognitive resources (Campanella et al., 2002). In addition, our results indicated that self-referential processing elicited smaller N2 amplitudes under negative emotional priming conditions than under neutral conditions in most electrodes. This is consistent with previous studies. Watson, Dritschel, Jentzsch, and Obonsawin suggested the existence of self-bias among the healthy adult group but not in the depressed group, whose members failed to connect emotional valence to the self (Watson et al., 2008). Ma and Han (2009) presented participants with their own faces and a friend’s face and found a self-face advantage in the low-threat context but a self-face disadvantage in the high-threat context. And Folstein and Van Petten (2008) focused on paradigms that elicited N2 components with an anterior scalp distribution. Specifically, the tasks they employed involved cognitive control, novelty, and sequential matching. Folstein and Van Petten (2008) argued that the anterior argued that the N2 belongs in the family of attention-related N2 components that, in the visual modality, have *a posterior* scalp distribution. These studies focused on the visual modality for which components with fronto central and more posterior scalp distributions can be readily distinguished. In this way, the results of the current work suggested that the threat messages of negative emotional pictures draw considerable attentional resources, which places self- referential stimuli in low level processing.

In the present study, highly self-relevant names elicited larger P3 amplitudes than moderately and non-self-relevant names. It demonstrated a significant degree of self-reference effect. Under different emotional priming conditions, results showed that self-referential stimuli could elicit significantly different P3 components. It is widely acknowledge that the novelty P3 is an index of the late phase of orienting response, which is sensitive to centrally controlled processes (Campanella et al., 2002; Carretié et al., 2004; Yuan et al., 2008a). The highly self-relevant stimuli received more in-depth and more sophisticated processing than moderately self-relevant stimuli. This suggested that the P3 component, unlike earlier P2 and N2 components, which reflected general processing of self-information, was an effective ERP index of the degree of self-reference effect. In addition, the current results indicated that self-referential processing elicited smaller P3 amplitudes under negative emotional priming conditions than under neutral conditions at most electrode sites. One possible explanation may be that the presentation of negative pictures initiated extended processing in the brain, attracted numerous attentional resources, and thereby might have weakened the degree of self-reference effect. In the N2 and P3 intervals, three sorts of names activated significant self-reference effects, and those effects peaked in midline sites.

For P2, N2, and P3 intervals, self-referential stimuli elicited smaller amplitudes under negative emotional priming conditions than under neutral conditions in central and central-parietal sites. Those outcomes are consistent with previous research. Chen J. et al. (2008) proposed that anterior cingulate cortex (ACC) and posterior cingulate cortex (PCC) at the midline site could be involved in self-referential processing. Fan et al. (2011) found self- referential stimuli induced more evident P3 amplitudes than familiar or unfamiliar stimuli, and this effect peaked at midline sites. From these superiority effects, we infer that the midline site could be critical to self-referential processing. After priming for negative emotional conditions, the midline site was obstructed, thus relegating a self-referential stimulus to low level processing. In addition, previous studies have reported that familiar stimuli evoke larger P3 amplitudes than unfamiliar stimuli (Bobes et al., 2000; Beauchemin et al., 2006; Fan et al., 2011). Stimuli that differ in degree of familiarity may be associated with changes in the degree of self-reference effect. But behavioral results have ruled out this possibility, because no significant difference in degree of familiarity was observed between all of the self-relevant stimuli and non-self-relevant stimuli. Hence, the P3 differences we found may be irrelevant to familiarity. Our results suggested that P3 could be positively correlated with the degree of self-relevance: the higher the degree of self-relevance, the larger the P3 amplitudes. While N2 has no such correlation with the degree of self-relevance, the P3 effect observed in present study should be ascribed to the extent of self-relevance instead of the other aspect of the self (Fan et al., 2013).

In general, the present study not only repeated the degree of self-reference effect, but also expanded upon previous studies. The current results demonstrate the influence of negative emotion on the degree of self-reference effect. Outcomes suggested that the degree of self-reference effect remain stable under neutral emotional condition. However, the degree of self-reference effect was weaker under negative emotional conditions, suggesting that the degree of self-reference effect is flexible. Specifically, negative emotional processing was found to weaken the degree of self-reference effect.

## Author Contributions

Conceived and designed the experiments: WF and YZ. Performed the experiments: WF, ZY, and JL. Analyzed the data: WF, ZY, and JL. Contributed reagents/materials/analysis tools: WF and RC. Wrote the paper: WF and XF.

## Conflict of Interest Statement

The authors declare that the research was conducted in the absence of any commercial or financial relationships that could be construed as a potential conflict of interest.
